# Deep-Learning-Based Approach for Iraqi and Malaysian Vehicle License Plate Recognition

**DOI:** 10.1155/2021/3971834

**Published:** 2021-11-06

**Authors:** Dhuha Habeeb, Fuad Noman, Ammar Ahmed Alkahtani, Yazan A. Alsariera, Gamal Alkawsi, Yousef Fazea, Ammar Mohammed Al-jubari

**Affiliations:** ^1^Institute of Sustainable Energy, Universiti Tenaga Nasional, Kajang 43000, Malaysia; ^2^Communication Engineering Department, Iraq University College, Basra, Iraq; ^3^School of Information Technology, Monash University Malaysia, Bandar Sunway 47500, Selangor, Malaysia; ^4^Department of Computer Science, College of Science, Northern Border University, Arar, Saudi Arabia; ^5^Department of Computer & Information Technology, Marshall University, 1 John Marshall Drive, Huntington, WV 25755, USA; ^6^NewTouch Smart Technology Solutions, Sana'a 51525, Yemen

## Abstract

Recognizing vehicle plate numbers is a key step towards implementing the legislation on traffic and reducing the number of daily traffic accidents. Although machine learning has advanced considerably, the recognition of license plates remains an obstacle, particularly in countries whose plate numbers are written in different languages or blended with Latin alphabets. This paper introduces a recognition system for Arabic and Latin alphabet license plates using a deep-learning-based approach in conjugation with data collected from two specific countries: Iraq and Malaysia. The system under study is proposed to detect, segment, and recognize vehicle plate numbers. Moreover, Iraqi and Malaysian plates were used to compare these processes. A total of 404 Iraqi images and 681 Malaysian images were tested and used for the proposed techniques. The evaluation took place under various atmospheric environments, including fog, different contrasts, dirt, different colours, and distortion problems. The proposed approach showed an average recognition rate of 85.56% and 88.86% on Iraqi and Malaysian datasets, respectively. Thus, this evidences that the deep-learning-based method outperforms other state-of-the-art methods as it can successfully detect plate numbers regardless of the deterioration level of image quality.

## 1. Introduction

Automatic vehicle license plate recognition (VLPR) has gained much popularity over the years with the rapid increase in various security and traffic applications. It is also a primary method that plays a crucial role in automatic monitoring, maintaining law enforcement on public roads, and controlling access to parking lots. Controlling border crossings and imposing speed limits for vehicles are two other applications of VLPR [1]. Image acquisition, license plate (LP) extraction, segmentation, and recognition are the steps involved in recognizing an LP. The acquisition of images is the first step in a VLPR system [1]. An LP is a rectangular metal plate with numbers, characters, and words that are permanently attached to the vehicle body and used to identify it [2]. Due to the fact that the plate is a dark spot of intensity and brightness within an area of the image, an identification system capable of extracting the captured images is required for vehicle number plate recognition [3]. Therefore, many methods, including deep learning (DL), support vector machine (SVM), and neural network (NN), are used to identify vehicle LPs. All of these methods are considered and discussed in this study [4–6].

**Figure 1:**
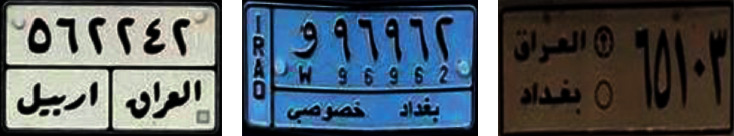
Samples of Iraqi license plates.

This study aims to look at different VLPR techniques available in both Arabic and Latin alphabets and compare the output of NN, SVM, and deep learning on Iraqi and Malaysian LPs. Before using VLPR, image preprocessing is required to achieve high accuracy. There are powerful libraries such as open VLPR which are promising; however, these libraries do not work well on Malaysian and Arab LPs because they are primarily designed for European and American plates. Regardless of vehicle type, Malaysian LPs have white characters, which vary greatly from European and American LPs. License plates in Arab countries, on the other hand, contain special Arabic letters, and certain countries use a combination of Arabic and Latin alphabets. To define the form of license or represent a region, most Arab countries use background colours or letters. This separates the Arabic-based LPs from those found in other nations, raising several problems during the learning process [7]. In Iraq, for example, the Arabic alphabet is used as an extension to plate numbers and also used to label the country, city, and type of vehicle. Iraqi vehicle LPs are classified into three categories, as seen in Figure 1. The first category (Figure 1(a)) contains the governorates of Iraqi Kurdistan (Erbil, Sulaymaniyah, and Duhok). The second pattern, seen in Figure 1(b), was in use prior to 2003. The third pattern, seen in Figure 1(c), is a recent pattern used in the central and southern governorates.

**Figure 2:**
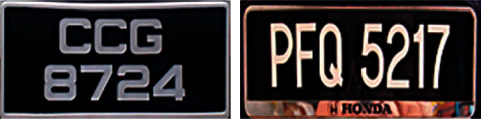
Samples of the Malaysian license plates.

Looking at the standards of Malaysian plate numbers, there is only one type, which has a black background with white fonts. As seen in Figure 2, the Malaysian LP includes letters and numbers in the Latin alphabet. In contrast to Iraqi plate numbers, this plate design makes identification even easier.

Several studies on LP recognition systems can be found in the literature. These analyses take into account the various characteristics of LPs, which vary by country in terms of colour, history, font, plate sizes, and language. The plates in Arab countries are noteworthy for featuring a combination of Latin and Arabic alphabets, necessitating thorough studies in the identification mechanism of such plates [3]. Few studies have adopted deep-learning approaches for Arab countries' plate number detection and recognition [8, 9]. Previous work has not taken into account the use of Iraqi plates of all governorates, including old and new styles. This is attributed to the fact that Iraqi plates come in a number of types and shades, with some having a white, red, or yellow background. As a result, Iraqi and Malaysian LPs are taken into account in this paper. While numerous studies have been carried out on Malaysian LPs [7, 10, 11], there have been very few studies on Iraqi LPs using conventional machine learning methods [12–14]. There has been no research into using deep learning for all Iraqi regions. However, a recent study addresses the Iraqi LP recognition for the Northern part of Iraq only [15].

In this study, to validate the robustness of the proposed approach, Arabic and Latin-based plate numbers are used (i.e., Iraqi and Malaysia plates). This research leads to the solution of this problem by using deep learning, and it contrasts the outputs of two conventional machine learning approaches, SVM and NN. The proposed method works for either Arabic or Latin alphabet plates. Deep learning does not usually need hand-crafted features as inputs, and it automatically learns to segment numbers from the LP in an end-to-end paradigm.

The following measures describe the general approach for this study: Firstly, vehicle photographs are collected from two separate nations, namely, Iraq and Malaysia. The data collection process is regulated by the same rules and regulations (e.g., image size, quality, and environment). Secondly, the steps of the recognition system are implemented using MATLAB software: The first step is the LP or region of interest (ROI) identification and plate field extraction from the vehicle. The second step is implementing LP segmentation, which involves separating letters, numbers, and words from the LP area. In the third step, the segmented plate numbers of the input vehicular LP images, which entail optical character recognition (OCR), are used by machine learning for classification into characters or numbers. Finally, the deep-learning approach is used to compare the outcomes of the Iraqi and Malaysian plates with two conventional machine learning approaches, namely, SVM and NN.

This paper is organized as follows: Section 1 presents the paper's introduction.  Section 2 presents the related works.  Section 3 presents the methodology and simulation.  Section 4 discusses experiments and results, and Section 5 concludes the paper.

## 2. Related Work

Previous studies on LP detection, segmentation, and recognition in various countries are discussed in this section. Then, we review selected previous studies and discuss the benefits and drawbacks of each method to determine the most important advantages in line with our proposed framework.

### 2.1. License Plate Detection

The plate detection step in ALPR is the first step after image acquisition. This is an incredibly critical step in assessing the system's performance. The plates are detected throughout six important detection approaches [16], as detailed in Section 1. (1) The first approach is the ability to distinguish the LP texture color transition, which is taken place between the background and letters. In [17], The texture function was discussed using a line of sharp vehicle tops that predicted a major change in the plate's grey hue. (2) Scanning the picture and checking for character variations are another approach. The plate area is determined by finding the letters/patterns in the image. (3) Boundary information, also known as edge features, is used, where rectangular shapes in the vehicle images can be searched for by scanning. As mentioned in [18], edge detection has been commonly used to evaluate the candidate rectangular regions and their positions. (4) In most countries, colour is used to detect LPs. To decide the area of the plate, the colour balance of LP and vehicle is calculated. (5) The fifth approach is defined by the characteristics of the global image. The image is scanned, and the resulting pixels are labelled depending on the pixel relation. As defined in [19], this is known as connected component analysis (CCA). It is more efficient than traditional procedures. (6) The sixth approach, known as miscellaneous attributes, analyses the image's structures and shapes using wavelet transformation technology. Some functions, such as CCA [19], are used because they are more effective than conventional procedures for rapidly processing the LP.

### 2.2. License Plate Segmentation

The second stage in the VLPR system is segmentation. Several techniques have been investigated and developed to break down the characters, including dynamic programming, hypothesis generation, CCA-based component analysis, projection, and modulation analysis. The projection analysis theory is the most widely used in many academic papers for the reason of its good scalability [20]. Valleys based on horizontal projection are used to achieve the upper and lower position of the characters. In [21], You-Only-Look-Once-Version-2 (YOLOV2) was used to detect LPs. However, only five types of mounting boxes are used for character recognition of LPs with oblique corners. In [22], the CCA method was used, where the surrounding boxes are collected using a two-hybridization process that segments the LP to distinguish the background from the foreground. The LP segmentation process is highly dependent on the dual coding, and irregularity of the plate affects the background texture, the poor contrast between the characters, the background, and the unevenness of the plate lighting.

In [23, 24], the CCA is used in character recognition and segmentation. However, some concerns about the performance were spotted. For example, the method failed to accurately identify some characters, that is, the presence of thin letters, which are difficult to handle and lead to a significant segmentation error. As a result, in order to prevent these defects, an algorithm is used to maximize the image's contrast, as has been used in several studies [25, 26].

### 2.3. License Plate Recognition

Recognition is the third step in the ALPR system. In the recognition phase, numbers and characters are automatically recognized from LP segments. To prevent plate numbers from being incorrectly recognized in the vehicle's image and vice versa, items in the plate are marked as letters or numbers depending on their position inside the plate [27]. The template matching system, for example, is used to identify different LPs in different countries. 2D and 3D prototype images are used, with cut letters and numbers rendered to the required pixel sizes. Some plates contain Latin alphabets, Arabic letters, and Arabic numbers as well. Letters and numbers are treated separately on the Latin alphabet plates, while Arabic plates are divided by country, letter, number, and country symbol. OCR equipment is used before character recognition. This is used to differentiate between physical LPs and electronic LPs. The precision with which these characters are segmented affects the accuracy of LP recognition. Many algorithms have been used in literature, including SVM [28], NN [29], and convolutional neural network (CNN) [9, 30, 31]. Although deep-learning-based methods have shown significant improvement in LP recognition, the computational cost of these methods is relatively expensive compared to other conventional machine learning methods.

### 2.4. Arabic License Plates System

Many Arab countries, including Iraq, Saudi Arabia, and Tunisia, have used LP identification, and several experiments have been performed to analyze this system in various ways [32].  Table 1 summarizes the findings of these experiments in terms of identification, segmentation, and recognition success rates.

Selmi et al. [31] studied deep learning on Tunisian LPs, which were characterized by different sizes and text, and the study was carried out in three steps: identification of the LP using Recurrent Neural Networks (RCNN), segmentation of the LP, and recognition of letters and numbers using groups of data (named Caltech and Application-Oriented LP (AOLP)). Due to the large volume of the used dataset, the results were 97.5% correct in identifying letters and numbers. Sakhawat et al. [33], on the other hand, used the CNN architecture, where analysis was performed, and handwritten numbers were studied. The proposed network was trained and proved to be successful in recognizing the handwritten characters.

In Omar et al. [9], data from the northern region of Iraq was used to study Iraqi LPs using a CNN model. At first, the dataset was collected, and then contrast was enhanced by image processing, followed by segmentation of plate number and country region and city area where the network was learned to classify. The results were very successful in identification, segmentation, and recognition with 92.10%, 94.43%, and 91.01%, respectively. Differently, Keesentini et al. [8], on the other hand, studied recognition of the Tunisian LPs using CNN, where the Tunisian plates were easily recognized because they only have one type, which consists of English numbers and the Arabic term (Tunisia). Despite the existence of environmental conditions, the proposed approach was stable, and the identification rate was 97.67% correct.

### 2.5. Latin License Plates System

Several algorithms have been developed in recent years, and many studies have been conducted on the essential methods used for detecting and identifying vehicles. As seen in Table 2, the detection, segmentation, and recognition of some recent LP studies from different countries were presented.

How and Sahari [7] reported that the deep convolutional neural network on Malaysian LPs showed the system was very effective for dealing with blurred images. Deep learning about Bangladeshi LPs was analyzed in multiple scenarios and settings [34]. In image analysis, the algorithm worked well. Furthermore, Miyata and Oka [35] performed machine learning research to expose Japanese LPs. This approach is focused on the shape of the LP and the details it provides, and although it has achieved positive results, it has certain shortcomings in the vehicle's context.

## 3. Methodology

This section outlines the procedures used in this study, including the details of the preprocessing and postprocessing measures for automated detection and identification of LP numbers. The new scheme made use of Iraqi and Malaysian LPs.

### 3.1. License Plates Dataset

An Iraqi vehicle LP is a plate used to identify Iraqi vehicles. The automated recognition system for Iraqi plates consists of three stages: detecting the plate, segmenting the plate, and finally recognizing the plate numbers. The Iraqi vehicle's plate includes several challenges that were resolved during this study because the Iraqi plate contains many patterns and colours, and the language used in plates is Arabic. In addition, the Iraqi plate has uppercase and lowercase characters, as well as Arabic and Latin alphabets, as seen in Table 3.

Malaysian LPs were inspired by the United Kingdom's LP concept in 1932. They were released after motor vehicles were brought under British control. Many vehicles with a common shape, like legal vehicles and heavy machinery, are classified in Table 4. Except for diplomats' and taxis' LPs, all plates in Malaysia have white characters on a black background on the front and back. The letters, numbers, and plate scale are all very well built as well.

The data collected in Iraq were taken using an iPhone 8 camera, and the images were processed by changing the size to dispose of unequal-size images. A total of 404 images were taken from the total Iraqi vehicles dataset. As for the Malaysian plates, 681 images were taken using a digital camera placed at the Universiti Putra Malaysia main gate entrance.

### 3.2. Proposed Methods

**Figure 3:**
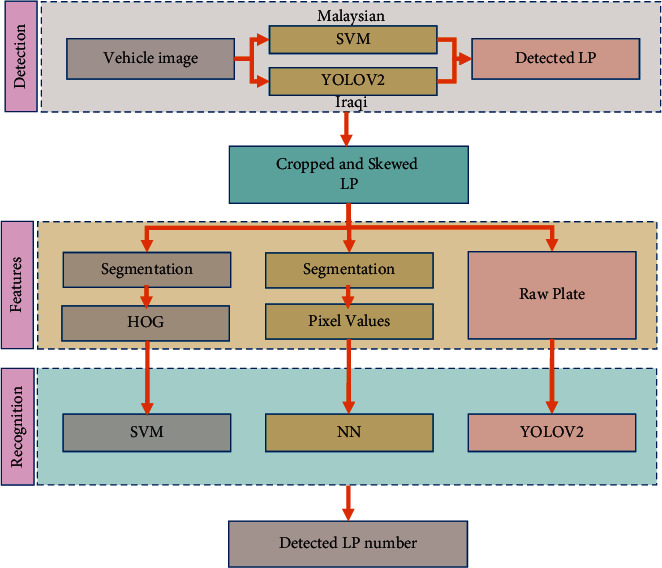
License plate detection/recognition system block diagram.

This research proposes a deep-learning-based algorithm for automated LP number identification and recognition. The YOLOV2 object detection CNN is used for the identification stage, which is then generalized for the recognition stage. This research aims to compare the efficiency of the proposed LP detection/recognition methods when dealing with plate numbers and characters based on Latin and Arabic.  Figure 3 depicts the process of LP detection/recognition.

Figure 3 outlines the LP detection/recognition protocol for both Malaysian and Iraqi plate numbers. The processing stages are summarized as follows:

- Given a complete captured vehicle picture with an LP, the YOLOV2 object detection for a deep-learning-based approach is used to detect the plate area
- The observed plate area is extracted or cropped and horizontal alignment is performed (skew correction) to facilitate plate number identification
- For Iraqi LPs, the single-step detection-recognition of YOLOV2 is used to measure the performance of detecting Arabic LPs
- Both Iraqi and Malaysian LPs are used to compare the success of using the deep-learning (i.e., YOLOV2) model in identifying the numbers/characters correctly, compared to traditional machine learning methods (i.e., SVM and ANN)

#### 3.2.1. License Plate Detection

The first step in this process is the identification of LPs. The collected LP image's resolution for Malaysian vehicles was 1232 pixels (height) and 2720 pixels (width), with a total plate size of 1.13 MB. However, Iraqi plates have a variety of designs and backgrounds in various colours. The identification process involves data training in order to find the best model and achieve the best outcomes. The YOLOV2 deep-learning-based model is used in this work, which is an architecture that learns deep representations from images using convolutional neural networks. This feature was first introduced by the scientist Joseph Redmond and Farhai, Santosh Devvala [36], and it has three versions, YOLOV1, YOLOV2, and YOLOV3.

For the purpose of supervised learning, we manually annotate (label) the Iraqi and Malaysian LP labels using the MATLAB Image Labeler Toolbox. Since this stage's goal was only to identify plate regions, the YOLOV2 object detection is equipped for a single class (plate) that can have varying sizes. The problem of multinorm plate areas is solved in YOLOV2 by using different anchor boxes during the candidate plate search process from a given vehicle picture. During the detection point, seven anchor boxes were calculated from the entire Iraqi and Malaysian plate dataset to be used in YOLOV2.

**Figure 4:**
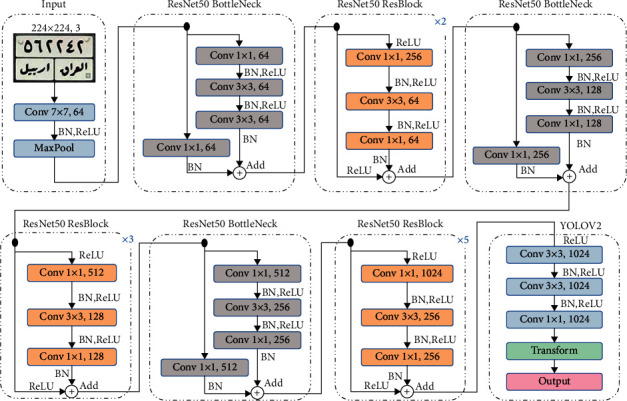
The architecture of the YOLOV2-based model and details of ResNet50 layers. The ResNet50 contains three main blocks, the input layers, the bottleneck layers, and residual (ResBlock) layers. For fine tuning, the pretrained deeper layers of ResNet50 are replaced with randomly initialized YOLO layers and trained again for LP segmentation and recognition.

YOLO is a supervised tool for detecting the LP area without the use of preprocessing or image enhancements. YOLOV2 uses separate fixed boxes to search each vehicle picture for the candidate LP region during the LP detection process. These boxes are known as network anchors, and they are sequentially handled one at a time. The CNN is then reinforced by YOLOV2 using backpropagation to extract meaningful feature maps from the LP region. The YOLOV2 properties can be summarized as follows: (1) it distinguishes itself by identifying and monitoring several objects of distinct frames; (2) it is a CNN with 24 feature extraction convolutional layers and two fully connected layers for classification; (3) GoogLeNet inspires it, but it differs in that it is more stable and has less trainable parameters [37].  Figure 4 depicts the YOLOV2 architecture [38].

The implemented model used the ResNet50 [39] as a backbone feature extraction network. The detection subnetwork is a small CNN compared to the feature extraction network and is composed of a few convolutional layers and layers specific for YOLOV2. We use the YOLOV2 layers on top of the pretrained ResNet50 to create a YOLOV2 object detection network. YOLOV2 layers require several inputs that parameterize a YOLOV2 network, that is, the network input size and anchor boxes. For the LP detection application in this paper, we constructed seven anchor boxes from the development dataset.

In YOLOV2, the convolutional grid is used to reduce the dimension of the input image, whereas other convolutional layers are used to extract features. Each grid frame is separated into *Sx* and *Sy*. The bound *B* squares are then searched, so each square has a different level of trust. This box output is denoted by the letter *p* (the object). As a result, each box consists of *x*,  *y*,  *w*,  *h*, and *c*, where (*x*,  *y*) denotes the centre of the box relative to the corresponding LP region, whereas (*w*,  *h*) denotes the width and height relative to the entire image, and *c* is the confidence score of this region.

#### 3.2.2. License Plate Segmentation

The method of extracting numbers from a given LP image, assuming this LP image was extracted from the vehicle image at the first processing step, the LP identification, is known as LP segmentation. YOLOV2 is used at this point to segment/extract the numbers of the Malaysian and Iraqi LPs. This is a recognition-based embedded mechanism. In other words, YOLOV2 can perform number segmentation as well as number recognition at the same stage. Other traditional machine learning techniques, on the other hand, enable the LP numbers to be segmented prior to the recognition point. In this regard, for traditional machine learning methods in this study, LP numbers were segmented using the MATLAB Image Labeler tool and then used to evaluate the recognition or classification of these segmented numbers. In segmentation, the LP image serves as the starting point from which the software should be able to collect uni-character images. The results obtained in this stage are used as inputs for the recognition stage and have excellent accuracy in plate identification. Furthermore, segmentation is another critical step in automated LP identification. If the partition fails, the outcome of plate recognition would be inaccurate, and provisional treatment of the plates is required for the segmentation to be accurate. For segments (plate numbers) classification, we first calculate the number of classes in the data used; for example, the letter “A” represents class one, and the letter “B” represents class two, and so on for the rest of the classes. The segmentation process can be summarized as follows: Firstly, the previously processed LP detection (Section 3.1) was used by the MATLAB Image Labeler module for manual plate numbers annotation/labelling; secondly, after all images have been labelled, the segmentation process is evaluated on the entire dataset (including training and testing subsets).

#### 3.2.3. License Plate Recognition Using SVM

A histogram of oriented gradients (HOG) is one of the most powerful features used to extract features from images [40]. Using a HOG provides better performance compared to other hand-crafted features [41]. The HOG is also called a shape-based feature that depends on the orientation graph's severity and is used through the segmented plate numbers in Section 3.2. In HOG, the image is divided into small and fixed groups to measure the orientation and gradient of pixels. The extracted features are used in machine learning to distinguish the different classes of plate numbers.

**Figure 5:**
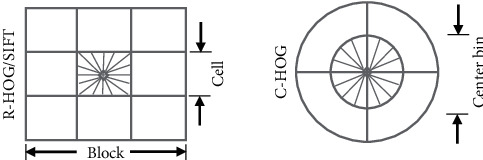
HOG feature extraction descriptors.

There are two forms of HOG descriptor, the R-HOG rectangular mass, and C-HOG circular mass, as in Figure 5. R-HOG consists of three parameters, the number of pixels per cell, the number of channels per graph, and the number of cells per block. One of the most important advantages of R-HOG blocks is that they are calculated in dense networks on a single scale without any directional alignment [42]. The circular C-HOG blocks are obtained by two variables, a variable that contains a central cell with a divided angle, and another that contains one central cell. These blocks contain four parameters: the expansion factor, the number of angled boxes, the number of diagonal boxes, and the radius of the central container [43].

In the recognition stage, two methods are used besides deep learning, that is, SVM and NN. The main idea is to use the extracted HOG features of the plate number for multiclass classification using SVM and NN classification methods. SVM is a statistical learning system that is widely used for character identification. The SVM system has proven to be successful in many types of research due to its superiority in a wide range of applications such as image classification and retrieval, movement detection during vehicle traffic, and recognition of written texts [44].

#### 3.2.4. License Plate Recognition Using NN

NN is one of the well-known machine learning techniques that can be used to recognize LPs. The input of the NN is a feature vector in which the NN leverages to learn more robust representations to map these features to the provided labels. In this study, the NN (or pattern recognition network) are feedforward networks that are composed of a single hidden layer that can be used for supervised classification tasks to classify input vectors according to target classes. We have used the MATLAB built-in NN model (i.e., patternnet function) and tested different hyperparameter configurations. For multiclass LP number classification application, the output of the NN is a matrix in which each row has 35 elements (categorical classes), out of which only one element is 1 and others are 0, denoting the chosen class [0–9, A–H, J–N, P–Y]. Letters and numbers are to be recognized through images that have been segmented in the previous stage.

At the recognition step, the numbers and letters from the segmented plates are recognized. The code is written in MATLAB software that invokes both the SVM classifier and the NN classifier. Five samples for each number/character were utilized for the training. The results of LP number recognition are saved in text files: one is for SVM results, and the other is for NN results. To calculate the LP recognition rate, the classification results are compared to the ground truth labels that contain the names of the correct plate numbers.

Similar to Malaysian plates, the recognition of the Iraqi LP is performed through two methods, namely, the SVM and NN. SVM uses the same feature that was applied for the Malaysian LP, HOG, that divides the image into small and fixed groups. The second method is the NN, which has 200 neurons.

#### 3.2.5. License Plate Recognition Using YOLOV2

YOLOV2-ResNet50, in its original design, accepts a minimum input image size of [224 × 224 × 3]. Throughout the training phase, the input image is split into *S* × *S* grid where each cell in the grid is responsible for predicting *B* bounding boxes. *B* is selected prior to the training phase, considering that each object has a different shape of anchor boxes. The convolutional layers of ResNet50 downsample the input image by a factor of 32; hence the output feature map will be the size of 224/32 = [*S* × *S*] = [13 × 13]. For example, if the number of objects to be detected from a given image is *C* = 10, the output of the YOLOV2 model is of 13 × 13 × *B* × 5 × *C*. Each predicted box has five components [*x*,  *y*,  *w*,  *h*, confidence] where (*x*,  *y*) denote the coordinates of the detected box centre point, (*w*, *h*) are the width and height, and a confidence score is a probability that the predicted box contains an object. In the testing phase, the YOLOV2 model uses the respective class-conditional probabilities to distinguish the predicted objects. The recognition phase is the last step in recognizing the Iraqi and Malaysian plates. The last stage in obtaining the image is followed by detecting and segmenting the plate. Recognition of the characters is obtained at the end of the segmentation phase of Iraqi and Malaysian plates. The VLPR system should read the vehicle image, output the character, and work with any data once the class number has been determined. Malaysian plates are identified by segmenting the plate into a letter and a number. As for the Iraqi plates, they contain several styles, but only three styles will be used. Through the image label feature, they will be segmented into letters, numbers, and names. Although the plates are of several types, with deep learning, these patterns are easily detected.

## 4. Experiments and Results

In this section, the results of the three methods of NN, SVM, and DL are presented. In addition, we conduct performance comparison and benchmarking of both machine learning and deep learning methods for Iraqi and Malaysian LP The performance evaluations are conducted using stratified 5-fold cross-validation to partition the subjects into the training, validation, and testing sets. The comparison is made in terms of accuracy, the volume of data, the feature extraction method, and the advantages and disadvantages of the three methods. The experiments are conducted using a computer with a built-in GPU and 16 GB of RAM memory.

For each experiment, we implement a stratified five-fold cross-validation for performance evaluations. The evaluations and comparisons of LP recognition performance were made by means of detection rate (classification accuracy) with details of misdetections and computational time. The recognition rate is used to measure the ratio of the total number of correctly recognized LP numbers divided by the total data volume. The LP detection (plate or number) results are also reported in terms of number of failures (no LP detected at all) or wrong detection (LP wrongly detected).

### 4.1. License Plates Detection Using YOLOV2

In this part, given a set of captured full images of vehicles, the results of the automatic plate detection are presented. In order to extract the LP regions, two independent YOLOV2 models are trained, one for Iraqi LPs and the other for Malaysian LPs. Each model was then evaluated on a hidden test set. Details of data partitions are shown in Table 5.

Tables 6 and 7 show the effectiveness of the trained models for the detection of LPs. The YOLOV2 model shows an average accuracy of 90.23% and 90.60% for the successful detection of LPs. The method shows stable performance across different folds and over both training and testing datasets, and in around 10% of testing set LP data were not correctly detected. The results demonstrate that the detection of Iraqi and Malaysian LPs is possible using the suggested end-to-end YOLOV2-based model.

### 4.2. License Plate Segmentation

**Figure 6:**
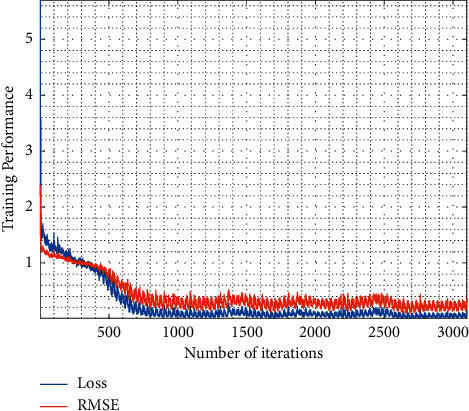
The learning curve as a function of training iterations (mini batches). Loss is the weighted losses of YOLOV2 objective function. RMSE is the weighted root mean square error of box segmentation regression.

Using the MATLAB Image Labeler tool, 30 classes were found in Iraqi dataset (i.e., Iraq, Baghdad, two, zero, six, eight, four, seven, Arbil, five, three, nine, R, Basra, private, one, A, F, fare, Dhi Qar, Maysan, Governmental, M, Sulaymaniyah, carry, W, B, Dhok, E, and N). On the other hand, Malaysian plates contain 35 classes (W, four, zero, nine, Y, B, G, U, three, six, eight, D, two, K, V, five, seven, P, N, L, M, S, C, one, A, Q, J, E, H, X, R, T, F, O, and Putrajaya). The labelled plate numbers were used for training the machine learning models for the task of plate number recognition, as detailed in the following subsections.  Figure 6 illustrates the training curve of the LP number segmentation process using YOLOV2 with decreasing loss and RMSE over iterations. The YOLOV2-ResNet50 was trained using Adam optimizer, with a learning rate of 0.001, 100 training epochs, and a batch size of 16.

### 4.3. License Plate Recognition Using SVM

**Figure 7:**
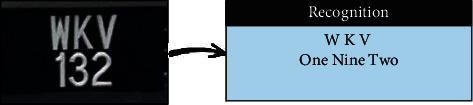
Malaysian result of wrong recognition (SVM).

**Figure 8:**
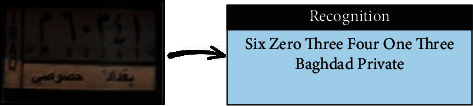
Iraqi result of wrong recognition (SVM).

The SVM was trained using HOG features extracted from the segmented plate numbers. As some LPs were not clear, they interfered with other vehicles. Figures 7 and 8 show some examples of LP numbers with inaccurate recognition, which could be due to the poor quality of photos, the distance between image and camera, or model overfit.

As shown in Table 8 (training- and testing-skewed columns), the number of images in each fold may vary from those reported in Table 5 because YOLOV2 was unable to correctly detect the LP region of some vehicle images. For SVM performance, the recognition rate for Malaysian LP numbers reached 91.92% for the training set and 88.70% for the testing set. As for Iraqi plates, as shown in Table 9, the recognition rate was 87.22% for the training set and 82.76% for the testing set, while the highest recognition rate of the testing set was observed at the second fold at 92.73%.

### 4.4. Licence Plate Recognition Using NN

**Figure 9:**
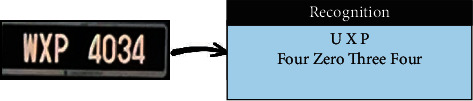
Malaysian result of wrong recognition (NN).

**Figure 10:**
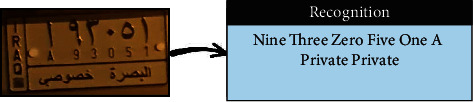
Iraqi result of wrong recognition (NN).

For NN evaluation, we tested different training functions, including Levenberg-Marquardt (trainlms), Bayesian regularization (trainbr), and scaled conjugate gradient backpropagation (trainscg). The number of neurons of the hidden layer is chosen from {100, 150, 200, 250, 300). The best performing combination of hyperparameters for LP recognition was found when using trainscg backpropagation with 200 neurons. Using HOG features, the NN was trained to recognize Malaysian and Iraqi LP numbers. Since the NN requires a larger amount of data to identify the plate, the recognition rate obtained was lower than that of the SVM. Figures 9 and 10 show two cases of LP numbers which could not be recognized by NN.

The results of NN recognition are summarized in Tables 10 and 11 for Malaysian and Iraqi LP numbers, respectively. The NN shows an average recognition rate of 88.58% for Malaysian LPs and 79.30% for Iraqi plates. Similar to SVM, the NN shows stable performance on Malaysian LP numbers. However, a poor performance was seen on Iraqi plates, reflecting the complexity of learning powerful patterns from LPs with diverse types/colours using a limited set of samples. As such, both SVM and NN show large variability in performance across folds when evaluated on Iraqi LPs.

### 4.5. License Plate Recognition Using YOLOV2

**Figure 11:**
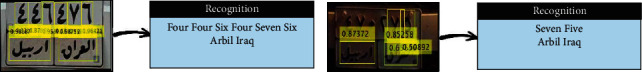
Iraqi result of (a) correct and (b) wrong recognition (YOLOV2).

**Figure 12:**
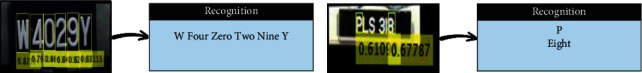
Malaysian result of (a) correct and (b) wrong recognition (YOLOV2).

A YOLOV2-based model was trained to recognize the Malaysian and Iraqi LPs. As an end-to-end deep-learning method, YOLOV2 leverages the powerful structure of convolutional layers to learn robust hierarchal representations from the LP images according to the given set of labelled boundary boxes. However, due to the deterioration in quality in some images, the YOLOV2-based model may fail to recognize certain LP numbers. Figures 11 and 12 show some examples of correct and wrong recognition of LP numbers using YOLOV2.

YOLOV2-based model results are shown in Tables 12 and 13. The recognition rate result for the Malaysian LPs on the hidden testing set reached 88.86%, with a maximum reported performance of 91.87% at the second fold. As for the Iraqi plates, the recognition rate of the testing set was 85.56%, with the highest rate of 92.86%, as can be seen in the fifth fold (Table 13). The results show that the YOLOV2-based model has a steady performance on both datasets. It is evident that there is a very small difference in the recognition rate between the two datasets, although Iraqi plates were more challenging in terms of diversity of colours and shapes. However, the YOLOV2-based model eliminates such problems as it works with all types of data. Thus, YOLOV2 has far exceeded the results of the previous two methods of SVM and NN, especially for Iraqi LPs.

### 4.6. Comparison

Table 14 compares the performance of the three LP number recognition methods in terms of the input feature set, average recognition rate on both training and testing sets, and the computation time.

The NN method has the lowest accuracy rate for Iraqi and Malaysian plates due to the possibility of the data being unbalanced in terms of number of labels and classes. Also, conventional machine learning methods performance is dependent on the amount and type of input features for a particular prediction task. In contrast, the YOLOV2-ResNet50, as a featureless approach, has the ability to automatically learn hierarchy of representations directly from the input images which explains the achieved higher performance. Also, performance is very low in Iraqi plates due to the fact that Iraqi plates contain more than one background and more than one pattern. In addition, the lack of sufficient samples of some letters, numbers, and names reduced the accuracy significantly. In general, all methods showed better performance on Malaysian compared to Iraqi LPs. This was because of the larger quantity of data that was available for Malaysian plates, which have only one pattern.

The SVM method outperformed the NN method in terms of LP number recognition. However, it was less successful than the YOLOV2-based approach due to difficulties encountered when working with several patterns for Iraqi plates. Furthermore, as the data count surpassed the feature count, the efficiency of the SVM method deteriorated. The main disadvantage of the SVM algorithm was in the classification result, which had some hyperparameters that needed to be tuned correctly for any given problem.

The YOLOV2-based model produced the highest accuracy rate compared to the other methods because of its ability to handle the LP data diversity, regardless of whether it contains a background in several colours or several patterns. In this study, the primary goal was to create a model that works on LPs with multiple patterns. This was possible when using the YOLOV2-based method, where, according to the results discussed above, (1) YOLOV2 achieved close performance on LPs with Arabic and Latin alphabet, which was not possible with the other two methods; (2) YOLOV2 works with big and repetitive data; (3) feature engineering in YOLOV2 is less complicated than the other two methods, since it mostly does not require hand-crafted features as input; (4) YOLOV2 automatically learns to segment the numbers out of the LP, but SVM and NN cannot do segmentation; as such, manual segmentation was made available to SVM and NN.

## 5. Conclusion

The aim of this paper was to use various machine learning techniques to classify and recognize Malaysian and Iraqi LP numbers. Three different machine learning methods were used for this reason (i.e., SVM, NN, and YOLOV2), and their identification and recognition success rates for both training and testing were compared. Like any other recognition scheme, this work encountered numerous issues, such as vehicle images with illuminance effects and varying sizes. For SVM and NN methods, the LP identification rate is proportional to the rate of LP numbers segmentation. As a result, if segmentation fails, the LP numbers recognition fails as well. This work deployed a YOLOV2-based method that relies on a deep-learning model for end-to-end LP numbers recognition to overcome these problems. An extensive comparison between the proposed deep-learning-based model with the traditional method (i.e., SVM and NN) was carried out, which included studying Malaysian LPs containing black background plates written in the Latin alphabet and Iraqi LPs with three different styles written in Arabic. The proposed method showed superior LP recognition performance with rates of 88.86% and 85.56% for Malaysian and Iraqi datasets, respectively. To further improve the performance of the proposed method, the authors suggest more data should be obtained for Iraqi LPs.

## Figures and Tables

**Table 1:** Summary of the previous works for Arabic license plates.

Ref.	Success rate (%)			Name of the country
	Detection	Segmentation	Recognition	
[31]	97.9	N/P	97.5	Tunisia
[70]	N/P	N/P	94.8	Saudi Arabia
[9]	92.2	99.3	99.3	Iraq
[8]	99.8	97.6	97.6	Tunisia

**Table 2:** Summary of the previous works for multilingual license plates.

Ref.	Success rate (%)			Name of the country
	Detection	Segmentation	Recognition	
[7]	N/P	N/P	95.89	Malaysia
[34]	N/P	98	98	Bangladesh
[35]	90	N/P	N/P	Japan

**Table 3:** Colours and types for Iraqi license plate.

Colour	Styles			Type
	Kurdistan region	Old style	New style	
White	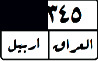	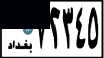	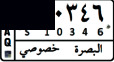	Private vehicle
Red	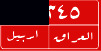	NA	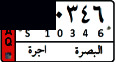	For bus and taxi
Blue	NA	NA	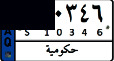	Governmental
Yellow	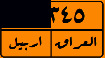	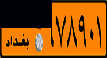	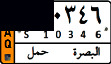	Trucks, tractors, and cranes
Orange	NA	NA	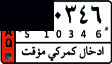	Temporary awaiting customs clearance

**Table 4:** Types of license plates of Malaysia.

Type of number plate (Malaysia)	Layout
Diplomatic	EX. 23-45-DC
Temporary	EX. A 2541 A (W/TP 2341 for KL)
Military	Ex. ZA 6789
Taxi	Private and commercial vehicles/Ex. HAB 2345
Private and commercial vehicles	EX. ABC 6789

**Table 5:** Result folds for Iraqi and Malaysian plates.

	Fold_1		Fold_2		Fold_3		Fold_4		Fold_5	
	Training	Testing	Training	Testing	Training	Testing	Training	Testing	Training	Testing
Iraq	314	90	332	72	327	77	329	75	314	90
Malaysia	540	141	555	126	549	132	544	137	536	145

**Table 6:** Detection results for Malaysian plates (YOLOV2).

	Data volume		LP detection				LP recognition	
	Total training images	Total testing images	Training set		Testing set		Training	Testing
			# of failures	# of wrongly detected	# of failures	# of wrongly detected	Detection rate	Detection rate
Fold 1	540	141	8	10	3	11	96.67	90.07
Fold 2	555	126	7	42	5	14	91.17	84.92
Fold 3	549	132	9	11	6	3	96.36	93.18
Fold 4	544	137	20	10	10	2	94.49	91.24
Fold 5	536	145	5	6	3	9	97.95	91.72
Average							95.33	**90.23**

**Table 7:** Detection results for Iraqi plates (YOLOV2).

	Data volume		LP detection				LP recognition	
	Total training images	Total testing images	Training set		Testing set		Training	Testing
			# of failures	# of wrongly detected	# of failures	# of wrongly detected	Detection rate	Detection rate
Fold 1	314	90	7	10	4	4	94.59	91.11
Fold 2	332	72	0	20	8	1	93.98	87.50
Fold 3	327	77	0	13	3	2	96.02	93.51
Fold 4	329	75	0	15	5	1	95.44	92.00
Fold 5	314	90	0	12	6	4	96.18	88.89
Average							95.24	**90.60**

**Table 8:** Recognition results for Malaysian plates (SVM).

	Data volume		LP detection		LP recognition	
SVM	Training-skewed	Testing-skewed	# training wrong	# testing wrong	Training results	Testing results
					Recognition rate	Recognition rate
Fold 1	522	113	53	10	89.85	91.15
Fold 2	473	104	30	16	93.66	84.62
Fold 3	486	115	37	12	92.39	89.57
Fold 4	502	120	40	13	92.03	89.17
Fold 5	504	118	42	13	91.67	88.98
Average					91.92	88.70

**Table 9:** Recognition results for Iraqi plates (SVM).

	Data volume		LP detection		LP recognition	
SVM	Training-skewed	Testing-skewed	# training wrong	# testing wrong	Training results	Testing results
					Recognition rate	Recognition rate
Fold 1	307	76	32	15	89.58	80.26
Fold 2	292	55	40	4	86.30	92.73
Fold 3	303	64	35	13	88.45	79.69
Fold 4	301	61	38	13	87.38	78.69
Fold 5	295	74	46	13	84.41	82.43
Average					87.22	**82.76**

**Table 10:** Recognition results for Malaysian plates (NN).

	Data volume		LP detection		LP recognition	
NN	Training-skewed	Testing-skewed	# training wrong	# testing wrong	Training results	Testing results
					Recognition rate	Recognition rate
Fold 1	522	113	13	11	97.51	90.27
Fold 2	473	104	6	14	98.73	86.54
Fold 3	486	115	2	10	99.59	91.30
Fold 4	502	120	3	14	99.40	88.33
Fold 5	504	118	0	16	100.00	86.44
Average					99.05	**88.58**

**Table 11:** Recognition results for Iraqi plates (NN).

	Data volume		LP detection		LP recognition	
NN	Training-skewed	Testing-skewed	# training wrong	# testing wrong	Training results	Testing results
					Recognition rate	Recognition rate
Fold 1	307	76	3	22	99.02	71.05
Fold 2	292	55	33	2	88.70	96.36
Fold 3	303	64	16	17	94.72	73.44
Fold 4	301	61	30	18	90.03	70.49
Fold 5	295	74	25	11	91.53	85.14
Average					92.80	**79.30**

**Table 12:** Recognition results for Malaysian plates (YOLOV2).

	Data volume		LP detection				LP recognition	
	# training images	# testing images	Training set		Testing set		Training	Testing
			# of failures	# of wrongly detected	# of failures	# of wrongly detected	Recognition rate	Recognition rate
Fold 1	522	127	7	15	9	9	95.79	85.83
Fold 2	473	107	0	5	5	9	98.94	86.92
Fold 3	486	123	10	4	2	8	97.12	91.87
Fold 4	502	133	1	9	4	9	98.01	90.23
Fold 5	504	133	0	7	4	10	98.61	89.47
Average							97.69	**88.86**

**Table 13:** Recognition results for Iraqi plates (YOLOV2).

	Data volume		LP detection				LP recognition	
	# training images	# testing images	Training set		Testing set		Training	Testing
			# of failures	# of wrongly detected	# of failures	# of wrongly detected	Recognition rate	Recognition rate
Fold 1	307	82	4	14	0	13	94.14	84.15
Fold 2	292	64	0	12	0	9	95.89	85.94
Fold 3	303	74	2	12	3	11	95.38	81.08
Fold 4	301	74	2	13	3	9	95.02	83.78
Fold 5	295	84	0	2	0	6	99.32	92.86
Average							95.95	**85.56**

**Table 14:** Comparison between SVM, NN, and YOLOV2.

	SVM		NN		YOLOV2	
	Iraq	Malaysia	Iraq	Malaysia	Iraq	Malaysia
Data size	404	681	404	681	404	681
Features	HOG	HOG	HOG	HOG	NA	NA
Testing acc.	82.76	88.70	79.30	88.58	85.56	88.86
Training acc.	87.22	91.92	92.80	99.05	95.95	97.69
Elapsed time	3.4 sec	6.9 sec	3.2 sec	6.6 sec	192.3 sec	155.6 sec

## Data Availability

The images data used to support the findings of this study are restricted by the respective authorities in order to protect the privacy of the car owners. Data are available from Ammar Ahmed Alkahtani (ammar@uniten.edu.my) for researchers who meet the criteria for access to confidential data.
